# Identification of Four Pathological Stage-Relevant Genes in Association with Progression and Prognosis in Clear Cell Renal Cell Carcinoma by Integrated Bioinformatics Analysis

**DOI:** 10.1155/2020/2137319

**Published:** 2020-03-28

**Authors:** Dengyong Xu, Yuzi Xu, Yiming Lv, Fei Wu, Yunlong Liu, Ming Zhu, Dake Chen, Bingjun Bai

**Affiliations:** ^1^Department of Colorectal Surgery, Sir Run Run Shaw Hospital, Zhejiang University School of Medicine, Hangzhou 310016, China; ^2^Department of Oral Implantology and Prosthodontics, The Affiliated Stomatology Hospital, Zhejiang University School of Medicine, Hangzhou 310006, China; ^3^Key Laboratory of Oral Biomedical Research of Zhejiang Province, Zhejiang University School of Stomatology, Hangzhou 310006, China; ^4^School of Medicine, Anhui University of Science and Technology, Huainan 232001, China; ^5^Department of Medical Oncology, Sir Run Run Shaw Hospital, Zhejiang University School of Medicine, Hangzhou 310016, China; ^6^Department of Nephrology, The First People's Hospital of Huzhou, The First Affiliated Hospital of Huzhou Teachers College, Huzhou, Zhejiang, China; ^7^Department of Urology, Wenzhou People's Hospital, Wenzhou, Zhejiang, China

## Abstract

Clear cell renal cell carcinoma (ccRCC) is a major histological subtype of renal cell carcinoma and can be clinically divided into four stages according to the TNM criteria. Identifying clinical stage-related genes is beneficial for improving the early diagnosis and prognosis of ccRCC. By using bioinformatics analysis, we aim to identify clinical stage-relevant genes that are significantly associated with the development of ccRCC. First, we analyzed the gene expression microarray data sets: GSE53757 and GSE73731. We divided these data into five groups by staging information—normal tissue and ccRCC stages I, II, III, and IV—and eventually identified 500 differentially expressed genes (DEGs). To obtain precise stage-relevant genes, we subsequently applied weighted gene coexpression network analysis (WGCNA) to the GSE73731 dataset and KIRC data from The Cancer Genome Atlas (TCGA). Two modules from each dataset were identified to be related to the tumor TNM stage. Several genes with high inner connection inside the modules were considered hub genes. The intersection results between hub genes of key modules and 500 DEGs revealed UBE2C, BUB1B, RRM2, and TPX2 as highly associated with the stage of ccRCC. In addition, the candidate genes were validated at both the RNA expression level and the protein level. Survival analysis also showed that 4 genes were significantly correlated with overall survival. In conclusion, our study affords a deeper understanding of the molecular mechanisms associated with the development of ccRCC and provides potential biomarkers for early diagnosis and individualized treatment for patients at different stages of ccRCC.

## 1. Introduction

Renal cancer is the deadliest urinary malignancy, with more than 350,000 cases worldwide [1]. Each year, over 140,000 people die from renal cancer, and the disease still has an increasing incidence [2]. Clear cell renal cell carcinoma (ccRCC), as the most common histologic subtype of renal cancer, can be clinically divided into four stages according to tumor size and the extent of invasion and metastasis [3, 4]. Currently, radiotherapy and chemotherapy are largely ineffective in the treatment of ccRCC, so surgery is the main treatment for most ccRCC, especially at the early stage [5, 6]. Unfortunately, most of the patients do not present any specific signs, and only 30% can be diagnosed during the early stage [7, 8]. For patients progressing to advanced stages, targeted therapies have been proposed as the most potential nonsurgical treatments because of their specificity and low toxicity [9]. Many targeted drugs have been approved for clinical use, while many others are undergoing clinical trials [10]. Immune checkpoint inhibitors with or without combination with tyrosine kinase inhibitors are the current standard of care. However, the median survival time of the treated patients still remains at a low level [11], which is far from satisfactory. Therefore, to improve the rate of early diagnosis and prognosis of ccRCC, it is necessary to comprehensively study the tumorigenesis and clinical stages of ccRCC and establish a relationship with more novel and specific biomarkers.

Originating from the proximal tubule, ccRCC showed abundant clear cytoplasm under the microscope because of deposition of lipid and glycogen, especially for larger tumors [12]. Although smoking [13], hypertension [14], and obesity [15] are considered risk factors, genetic variation also plays a critical role during the tumorigenesis process. Some specific gene mutations and corresponding signal pathways have already been proven to be closely associated with ccRCC [16]. Nearly 90% of ccRCC is characterized by the aberration of VHL [17], while PBRM1 is considered the second major tumor suppressor gene in ccRCC [18]. Previous studies have revealed a correlation between the lower expression of VHL and PBRM1 and a higher Fuhrman grade [19]. BAP1 is another tumor suppressor in ccRCC [20, 21], the low expression of which is significantly associated with high grade but not survival [22]. However, another study has indicated that loss of BAP1 expression suggests poor prognosis in metastatic ccRCC [23]. Therefore, dynamic changes in genes in different stages are of great importance in the occurrence and development of ccRCC, as well as the treatment and prognosis of this disease. Notably, a great difference remains in prognosis depending on whether the disease is diagnosed earlier or later. The 5-year overall survival rate is 92% if diagnosed in stage I but drops sharply to 23% in stage IV. Thus, identifying clinical stage-related genes is beneficial for improving the early diagnosis and prognosis of ccRCC.

Currently, bioinformatics analysis is becoming a useful approach to identify relevant genes to certain diseases. Weighted gene coexpression network analysis (WGCNA) [24] has emerged as an effective method for analyzing gene expression data and to discover the relationship between gene clusters and tumor phenotypes. Several researchers have applied this approach to screen the genes involved in the genesis of ccRCC [25–29]. They take the understanding of the molecular mechanisms of ccRCC a step further. However, precise and efficacious molecular targets for the treatments of ccRCC have not been found. Thus, identifying novel therapeutic targets or biomarkers is still a priority for diagnostic or prognostic applications.

In this study, we aim to more precisely identify clinical stage-related differentially expressed genes (DEGs) that are significantly associated with the occurrence and development of ccRCC by applying integrated bioinformatics analysis. We analyzed a total of 261 raw data files from GSE53757 and GSE73731, then divided the data into five groups and compared the gene expression of normal tissue with ccRCC stages I, II, III, and IV. As a result, we identified 500 common DEGs that were either upregulated or downregulated in each stage of ccRCC. Furthermore, functional enrichment analyses were performed and protein-protein interaction (PPI) networks were constructed so as to explore the biological roles of those DEGs. Moreover, to obtain DEGs highly correlated with the stage of ccRCC, WGCNA was applied to detect the modules and hub genes associated with the tumor stage in two independent data sets: The Cancer Genome Atlas Kidney Renal Clear Cell Carcinoma (TCGA-KIRC) data and GSE73731. Finally, the intersection results between the hub genes from key modules and 500 DEGs showed UBE2C, BUB1B, RRM2, and TPX2 as key hub genes that were highly associated with the clinical stages of ccRCC. qPCR and the Human Protein Atlas database were utilized to validate the roles of key hub genes both at the RNA expression level and at the protein level. Survival analysis showed that these 4 genes were all significantly related to overall survival. In short, UBE2C, BUB1B, RRM2, and TPX2 could probably be potential biomarkers for early diagnosis and individualized treatment for ccRCC.

## 2. Materials and Methods

### 2.1. Screening and Preprocessing of Microarray Data

All data in this manuscript were collected under the guidelines approved by the First Affiliated Hospital of Zhejiang University School of Medicine's institutional review board and complied with the current laws in China. Neglecting language, race, region, and time constraints, we systematically researched the Gene Expression Omnibus (GEO) database (https://www.ncbi.nlm.nih.gov/geo/) with several defined strategies: (1) tissue source was ccRCC and/or paracancerous tissue, (2) the Affymetrix platform was GPL570, (3) data sets had staging information, and (4) the tissue came from patients who did not receive any antitumor treatments. The gene expression profiles of GSE53757 and GSE73731 were downloaded and analyzed by the affy package [30] in R software (R version 3.6). Relative logarithmic expression (RLE) and normalized unscaled standard errors (NUSE) [31] were used to evaluate the quality of the data. We fitted a probe-level model to the data and created two plots for the dataset. The deviant arrays can be identified by their not being centered at 0 in the RLE boxplot or 1 in the RUSE boxplot or being more spread out than the other arrays. After excluding the outliers, we performed a standard robust multiarray average (RMA) [32] procedure to create an expression matrix. During the process, the raw intensity values were background-corrected, log2-transformed, and then quantile-normalized.

### 2.2. Identification of DEGs

Raw data files contained in GSE53757 and GSE73731 were divided into five groups according to their tissue source and stage: ccRCC stages I, II, III, and IV and normal tissues. The gene expression levels in ccRCC stages I, II, III, and IV were compared with those in normal tissue using the limma package [33] in R software. Those genes with the cut-off criteria of ∣log2FC∣ > 2 (FC: fold change) and an adjusted *P* value < 0.05 [34] were considered DEGs, which were further divided into upregulated and downregulated genes.

### 2.3. Functional Enrichment Analysis

To explore the biological functional roles of DEGs associated with ccRCC, Gene Ontology (GO) and Kyoto Encyclopedia of Genes and Genomes (KEGG) pathway enrichment analyses were performed by using the clusterProfiler package [35] (http://bioconductor.org/packages/release/bioc/html/clusterProfiler.html) in R software. Under the condition of *P* < 0.05 and *q* < 0.02, significant biological process (BP), molecular function (MF), cellular component (CC) terms, and KEGG pathways were selected and visualized.

### 2.4. PPI Network Construction

The online database STRING [36] (version 11; https://string-db.org/) performed PPI networks of DEGs, which covered a total of 9,643,763 proteins from 2,031 organisms. DEGs were uploaded to STRING, and the results were visualized in Cytoscape software [37] (version 3.7.0; http://www.cytoscape.org/) with the minimum required interaction score of 0.700. In Cytoscape, dysregulated genes were plotted in different colors.

### 2.5. Data Collection and Preprocessing for WGCNA

Raw RNA sequence data and corresponding clinical information of KIRC patients were downloaded from TCGA. The data were concatenated to a matrix with gene symbols as row names and TCGA patient barcodes as column names. Then, we removed the control samples and samples with incomplete clinical information. Genes with zero counts in more than 80% of samples were also excluded. We normalized the matrix by the voom function [38] from the limma package [39], as RNA-seq read counts usually follow a negative binomial distribution. In addition to TCGA data, we also performed WGCNA on the normalized GSE73731 data. Finally, we chose the top 75% of the variant genes for WGCNA. In detail, the median absolute deviation (MAD) was used as a robust measure of variability.

### 2.6. Weighted Gene Coexpression Network Analysis

Weighted gene coexpression network analysis was performed using the WGCNA package in R [24]. The first step in this process was to calculate a similarity matrix using biweight midcorrelation [40], as it is more robust to outliers. After that, a weighted adjacency matrix was defined by raising the coexpression similarity to an appropriate soft-thresholding power. We chose the best power (*β*-value) based on the criterion of approximate scale-free topology [41]. Then, to minimize the effects of noise and spurious associations, we transformed the adjacency into a topological overlap matrix (TOM) and calculated the corresponding dissimilarity [42]. We now used hierarchical clustering to produce a hierarchical clustering tree and dynamic tree cut method to assign coexpressed genes to each module [43]. Modules were constructed with a minimum module size of 30 genes, and highly similar modules were combined using a dissimilarity threshold of 0.25.

### 2.7. Identification of Stage-Related Modules

Along with the module identification procedure, the module eigengenes were generated by principal component analysis (PCA). We used these module eigengenes and external clinical parameters to perform a module-trait relationship (MTR) analysis. The Pearson correlation coefficient and *P* value were calculated in this process. A heat map was drawn to present the results so that we could easily identify which modules related to the tumor stage. We also measured gene significance based on the correlation of a gene expression profile with a sample trait, and the module significance was the average absolute value of the gene significance measure for all genes in a given module. A barplot of the module significance for all modules detected was drawn. The highest module had the strongest correlation with the clinical trait.

### 2.8. Module Preservation Analysis

Sometimes, the modules identified are not reproducible in another dataset with similar samples, which means the quality of the modules is poor. Module quality measures based on density and separability measures can be used to confirm that the reference modules are well defined [44]. To verify whether the modules we selected are preserved, we utilized the modulePreservation function in the WGCNA package to calculate module preservation statistics between GSE73731 and an independent data set, GSE53757.

### 2.9. Identification of Hub Genes

After the construction of the network and the identification of stage-related modules, we explored individual genes within the coexpression module. The hub genes were selected on the basis of the module membership and gene significance. Module membership is measured by the correlation between the profile of the gene and the eigengene of the module, which describes how closely related the gene is to the module. Criteria for selecting the hub genes were as follows: module membership > 0.8 and gene significance > 0.2. Finally, we obtained the intersection of these hub genes and DEGs for further analysis.

### 2.10. Patient and Tissue Sample Collection

This study was approved by the Sir Run Run Shaw Hospital and Zhejiang University Ethics Committee (No. 20100823). All patients involved in this study had informed consent. Fresh cancerous tissue samples were obtained directly from the operation specimens of 8 patients who had undergone surgical resections for the kidney at the Department of Urology, Sir Run Run Shaw Hospital, Hangzhou, Zhejiang, China, between September 2010 and March 2011. Written informed consent for tissue collection was obtained from all patients prior to their surgical procedures. The adjacent normal tissues were collected from more than 5 cm away from the cancerous tissue.

### 2.11. qPCR Validation of Key Hub Genes

The overlapping genes in hub genes and DEGs were defined as key hub genes. We validated their expression levels by qPCR. Following the manufacturer's instructions, the RNA of tissue samples was extracted using a TRIzol reagent (Invitrogen; Thermo Fisher Scientific, Inc., Waltham, MA, USA). RNA was quantified by using a NanoDrop 2000c spectrophotometer (Thermo Fisher Scientific, Inc., Waltham, MA, USA). cDNA was synthesized utilizing an RNeasy Mini Kit (Takara, Kyoto, Japan). qPCR analysis was executed with a SYBR Green Master Mix (Takara) with a program of 95°C for 5 min, 45 cycles of 95°C for 5 sec, and 60°C for 30 sec; 1 cycle of 95°C for 5 sec, 60°C for 1 min, and 95°C for 15 sec; and, finally, 50°C for 30 sec. The 2-*ΔΔ*Cq method was used to analyze relative expression. Expression of mRNAs was normalized to *β*-actin. The primers were designed with the online tool (https://www.genscript.com/tools/real-time-pcr-tagman-primer-design-tool) and synthesized by Shanghai Generay Biotech Co. Ltd. (Shanghai, China). Primers are listed in Table 1.

### 2.12. Validation and Survival Analysis of Key Hub Genes

To validate both the RNA expression level and the protein level of hub genes, the website server GEPIA (Gene Expression Profiling Interactive Analysis) [45] and the Human Protein Atlas database [46] were utilized. With the help of the GEPIA website, the relative RNA expression level between ccRCC tissue and normal renal tissue was visualized with box plots. The Human Protein Atlas database was used to map the proteins in the tissues. Finally, the survival analysis of ccRCC patients was performed using TCGA data from the GEPIA website as well.

## 3. Results

### 3.1. Basic Characteristics of Microarray Data

**Figure 1:**
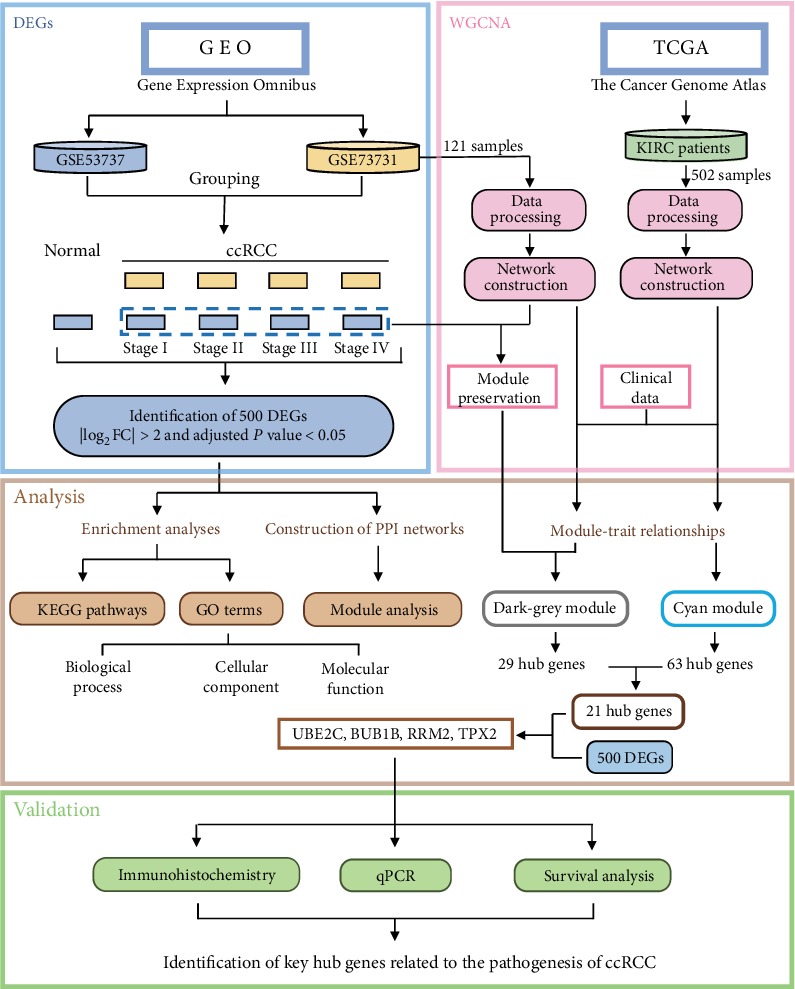
The flow chart of our study.

The workflow of our study is shown in Figure 1. On the GPL570 ([HG-U133_Plus_2] Affymetrix Human Genome U133 Plus 2.0 Array) platform, we screened out two data sets (GSE53757 and GSE73731) with pathological stage information. Each data set had data from more than 50 patients. All raw data files were assessed with the RLE and NUSE algorithms. Finally, excluding the data files that did not satisfy the requirements, a total of 261 raw data files were filtered out. These data were divided into five groups: ccRCC stages I, II, III, and IV and normal tissues. The normal tissue group had 72 raw data files, and ccRCC stages I, II, III and IV had 62, 29, 41, and 57 raw data files, respectively (Table 2).

### 3.2. Identification of DEGs

**Figure 2:**
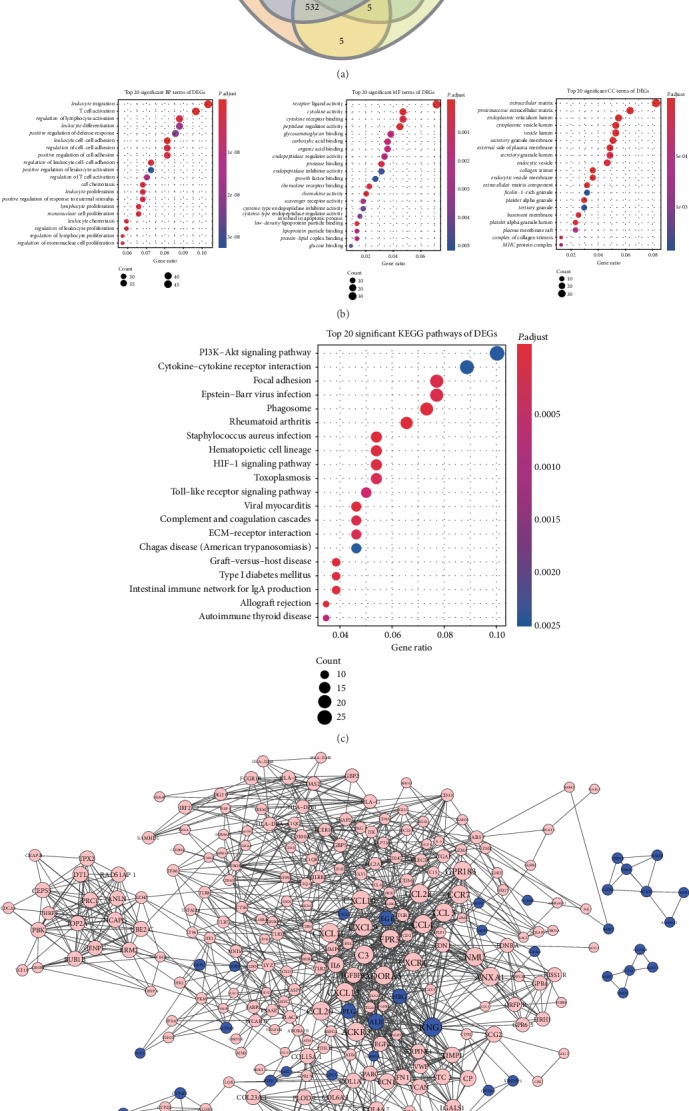
Identification and functional enrichment analyses of 500 DEGs. (a) Constructing a Venn diagram in four comparison groups with the cut-off criteria of ∣log2FC∣ > 2 and an adjusted *P* value < 0.05. (b) GO enrichment analysis showing the top 20 terms of the BP, MF, and CC categories. (c) Top 20 significant KEGG pathways. (d) PPI networks of 500 DEGs. Pink nodes indicate upregulated DEGs and blue nodes indicate downregulated DEGs.

The gene expression levels of the ccRCC stage I, II, III, and IV tissues were compared with those of normal renal tissues separately. After background correction, normalization, and logarithmic conversion by conducting RMA, we obtained 1410 DEGs between normal tissue and ccRCC stage I, 632 DEGs between normal tissue and ccRCC stage II, 1915 DEGs between normal tissue and ccRCC stage III, and 1388 DEGs between normal tissue and ccRCC stage IV with the cut-off criteria of ∣log2FC∣ > 2 and an adjusted *P* value < 0.05. Then, as shown in Figure 2(a), 500 common DEGs were extracted from the 4 comparison groups. Among these 500 DEGs, 407 genes were upregulated and 93 genes were downregulated.

### 3.3. Functional Enrichment Analyses of DEGs

With the clusterProfiler package in R software, we conducted GO and KEGG pathway enrichment analyses to shed new light on the functions of the identified DEGs. We uploaded the lists of DEGs, and the results of the GO analysis showed that these genes were enriched in different functional categories: BP, MF, and CC. The top 20 significant terms of each functional category are plotted in Figure 2(b). Most of the DEGs were enriched in “leukocyte migration,” “receptor ligand activity,” and “extracellular matrix” terms. Regarding the KEGG pathways, most of the DEGs were associated with the “PI3K-Akt signaling pathway” and “cytokine-cytokine receptor interaction,” which was consistent with the biological process terms (Figure 2(c)).

### 3.4. Construction of PPI Networks

PPI networks of DEGs were built in accordance with the online database STRING and visualized in Cytoscape software with the minimum required interaction score of 0.700. The PPI networks of 500 DEGs were composed of 250 nodes and 1214 edges after excluded lowest clustering score nodes (Figure 2(d)). Pink nodes indicated upregulated DEGs, while blue nodes indicated downregulated genes.

### 3.5. Weighted Gene Coexpression Network Analysis Using GSE73731

The WGCNA was performed to analyze the GSE73731 microarray data and the TCGA RNA-seq data. Both datasets were properly normalized and filtered to reduce outliers.

**Figure 3:**
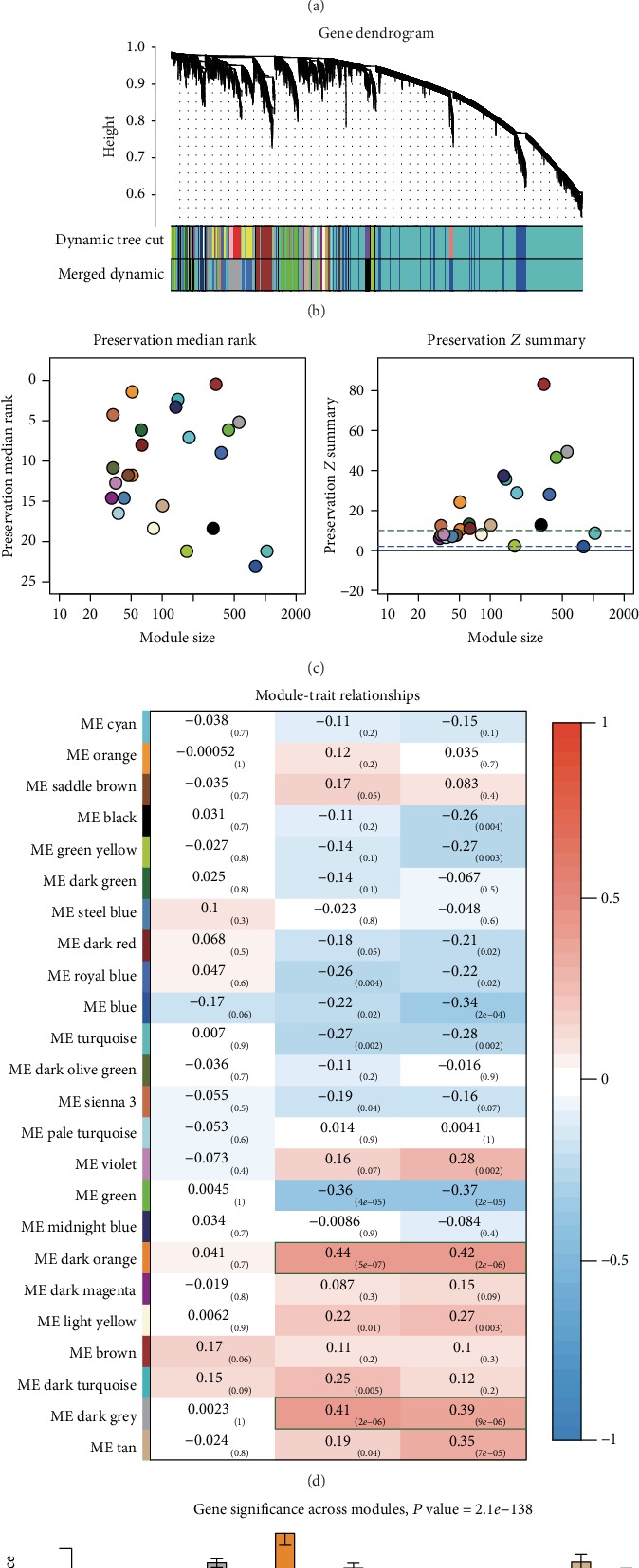
Weighted gene coexpression analysis for GSE73731. (a) Analysis of the scale-free fit index and mean connectivity for various soft-thresholding powers. (b) The clustering dendrogram based on the dissimilarity (1-TOM) using the dynamic tree cut method. (c) Module preservation test with an independent dataset, GSE53757. (d) Module-trait relationship heat map based on the Pearson correlation coefficient between module eigengenes and clinical parameters (age, grade, and stage). (e) Barplot for average gene significance across modules associated with the pathological stage for ccRCC.

After data preprocessing, 121 samples with complete clinical information and 9420 varying genes were analyzed in the GSE73731 dataset by WGCNA. Constructing a weighted gene network entailed the choice of the soft-thresholding power to which coexpression similarity was raised to calculate adjacency. Using the pickSoftThreshold function in the WGCNA package, we selected 3 as the best *β* for the following analysis (Figure 3(a)). As we assigned all of the genes to their corresponding modules, 23 modules were detected in the GSE73731 dataset (Figure 3(b)). According to the module-trait heat map (Figure 3(d)), we discovered that the dark-orange and dark-gray modules were most related to tumor stage as well as tumor grade. In addition, Figure 3(e) showed that the gene significance with the tumor stage of these two modules was also the highest across modules. The sequence preservation test indicated that the dark-gray module was well preserved in the GSE53757 dataset; however, the dark-orange module was preserved (Figure 3(c)). Considering that the dark-orange module had a relatively small size and poor preservation, we chose the dark-gray module as our target module that contained 553 genes in this module.

### 3.6. Weighted Gene Coexpression Network Analysis Using TCGA-KIRC Dataset and Filtration of Hub Genes

**Figure 4:**
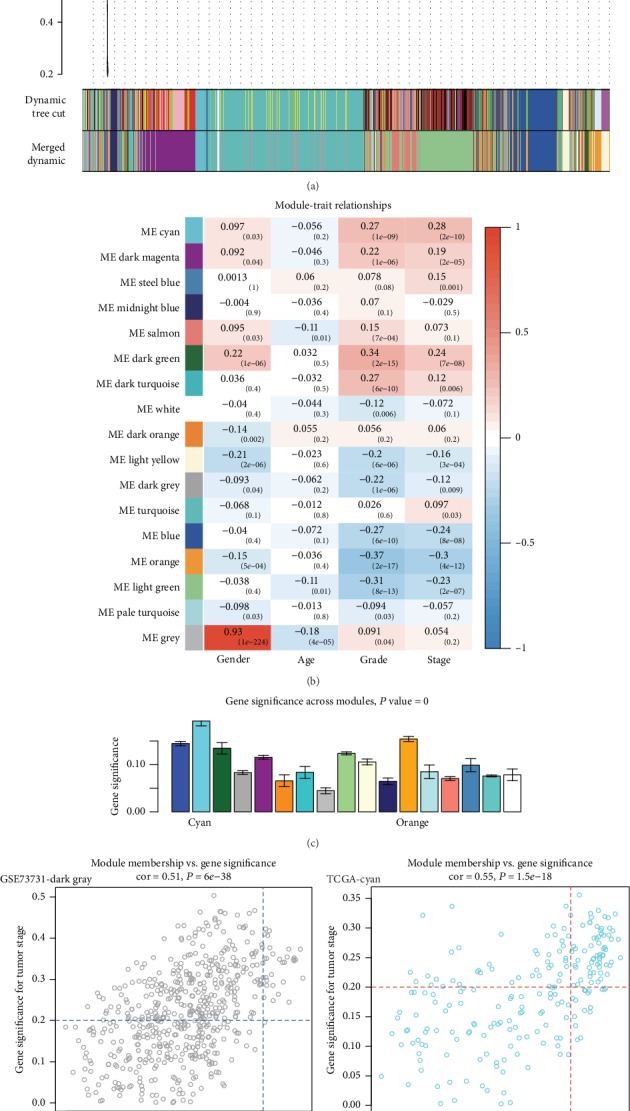
Weighted gene coexpression analysis for TCGA and screening for intramodular hub genes of key modules. (a) The clustering dendrogram based on the dissimilarity (1-TOM) using the dynamic tree cut method. (b) Module-trait relationship heat map based on the Pearson correlation coefficient between module eigengenes and clinical parameters (gender, age, grade, and stage). (c) Barplot for average gene significance across modules associated with the pathological stage for ccRCC. (d) Scatter plot of module eigengenes in the dark-gray module from GSE73731. (e) Scatter plot of module eigengenes in the cyan module from TCGA. Dots in the upper-right corner in both plots represent hub genes in the module.

Since the GSE73731 dataset had relatively few data with a clinical stage phenotype, we used the TCGA-KIRC dataset to verify the hub genes by constructing another coexpression network. With the TCGA-KIRC dataset, the network was composed of 12,000 gene expression profiles derived from 502 KIRC tumor tissues. Following the above procedure, we clustered genes into 16 different modules (Figure 4(a)) and determined that the cyan module was most related to the tumor stage phenotype (Figures 4(b) and 4(c)). Using the criteria mentioned earlier, we filtered out 29 hub genes (Figure 4(d)) in the dark-gray module with high module membership and gene significance. With the same criteria, 63 genes in the cyan module were identified as hub genes (Figure 4(e)). When we combined the hub genes from the GSE73731 and TCGA-KIRC data, 21 genes were found in common. Finally, we took the intersection of the common hub genes and DEGs from the previous section. Four genes that were identified as DEGs were also presented among the common hub genes (Table 3).

### 3.7. Validation of RNA and Protein Expression Level for Key Hub Genes

**Figure 5:**
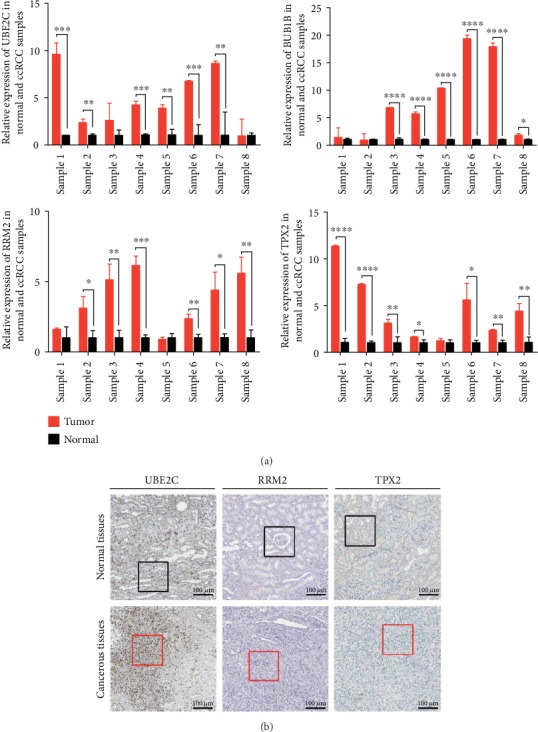
Validation of RNA and protein expression levels for hub genes. (a) Experimental validation of UBE2C, BUB1B, RRM2, and TPX2 in 8 paired clinical ccRCC samples. Data are presented as the means ± SEM. ^∗^*P* < 0.05, ^∗∗^*P* < 0.01, ^∗∗∗^*P* < 0.001, and ^∗∗∗∗^*P* < 0.0001. (b) UBE2C, RRM2, and TPX2 protein expression levels in ccRCC as compared to those in normal kidney tissues by IHC staining. Scale bars represent 100 *μ*m. Black and red square boxes represent typical normal kidney and ccRCC tissues, respectively.

Table 4 showed the histopathological characteristics of ccRCC patients. Experimental validation of UBE2C, BUB1B, RRM2, and TPX2 was successful in 8 paired clinical ccRCC samples, demonstrating that all of them were upregulated in ccRCC (Figure 5(a)). As for the protein expression level's validation, it was assessed to indicate that UBE2C and TPX2 expressions were significantly higher in tumor tissues than in normal tissues in accordance with the Human Protein Atlas database (Figure 5(b) and Table 5). Besides, it could be seen from the microscope's photographs of IHC staining that there was a clear boundary between the cells of normal kidney tissues, while the cell morphology of kidney cancer tissues exhibited diversities, and nuclear heteromorphs could be observed as well.

### 3.8. Validation and Survival Analysis of Key Hub Genes

**Figure 6:**
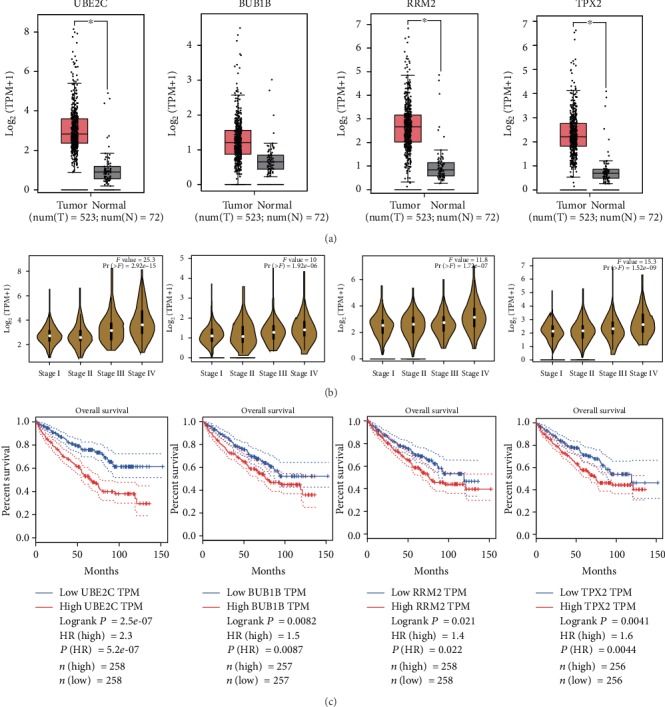
Validation of hub genes in the TCGA dataset. (a) UBE2C, BUB1B, RRM2, and TPX2 expressions in 523 KIRC patients compared with 72 normal samples. (b) Assessing hub genes' expression at different stages. (c) Overall survival curves of UBE2C, BUB1B, RRM2, and TPX2.

The RNA expression levels of UBE2C, BUB1B, RRM2, and TPX2 were also validated in the TCGA dataset. With the cut-off criteria of ∣log2FC∣ > 1 and *P* < 0.01, UBE2C, BUB1B, RRM2, and TPX2 were upregulated in 523 ccRCC samples as compared to 72 normal samples (Figure 6(a)). Meanwhile, pathological stage plots showed that UBE2C, BUB1B, RRM2, and TPX2 were significantly correlated with the stage of ccRCC (Figure 6(b)). These genes were more abundantly expressed in advanced kidney cancer as compared to early ccRCC, which was consistent with the above analysis. In Figure 6(c), we analyzed the overall survival (OS) of UBE2C, BUB1B, RRM2, and TPX2 in ccRCC. The lower expression levels of hub genes predicted better prognosis of ccRCC than higher expression.

## 4. Discussion

To explore the occurrence and development of diseases from a novel perspective, bioinformatics analysis is becoming an emerging and effective technique [47, 48]. Clear cell renal cell carcinoma is a major histological subtype of renal cell carcinoma, which is one of the deadliest forms of urinary malignancy [1]. Sequential changes in gene expression in different clinical stages play essential roles in ccRCC. In recent decades, strides have been made in identifying pathogenesis-related genes and therapeutic potential biomarkers of ccRCC [26, 49, 50]. However, most of them only look into how genes were dysregulated between cancerous and paracancerous tissues. Our study has a more detailed grouping, as there are five comparison groups in total. The gene expression levels in ccRCC stages I, II, III, and IV are compared with those of normal renal tissues. More precisely, we filter four genes highly related to clinical stages by WGCNA and multidimensional validations.

In our study, we downloaded the gene expression profiles of GSE53757 and GSE73731 and screened 500 DEGs involved in the development of ccRCC. GO terms of BP revealed that most of the DEGs were enriched in inflammation- and immunity-related categories. KEGG pathway analysis revealed that the DEGs were mainly involved in the “PI3K-Akt signaling pathway,” “cytokine-cytokine receptor interaction,” and “focal adhesion” pathways. The above results further suggest that ccRCC is a disease associated with cytokinesis and inflammation [51, 52], providing bioinformatics evidence for deep research. Moreover, aiming to make our results more accurate, WGCNA was performed in GSE73731 to identify gene coexpression modules related to clinical stages of ccRCC, and the modules were preserved in GSE53757. The dark-gray module was confirmed, and hub genes were derived from this module. By the same token, TCGA-KIRC data were also downloaded to perform WGCNA, and the corresponding results were obtained.

After that, we obtained 4 key hub genes of interest that overlapped between 500 DEGs and 21 common hub genes selected in the GSE73731 and TCGA-KIRC data by WGCNA, including UBE2C, BUB1B, RRM2, and TPX2. Although previous studies have reported the association of these genes with ccRCC, most of them either used simple methods or lacked experimental verification, reducing the credibility of their results. For instance, overexpressed RRM2 was experimentally confirmed to be associated with a trend toward the advanced pathological stage, high Fuhrman grade, and poor prognosis [53]. In addition, a recent study reported that miR-99a-3p could regulate RRM2 in sunitinib-resistant ccRCC, showing a potential antitumor effect [54]. Analyzing TCGA and tissue microarray data showed that TPX2 was associated with advanced grade and stage of ccRCC, which could be a potential therapeutic target as well [55]. These results are consistent with ours. As for UBE2C and BUB1B, Yuan et al. reported that UBE2C and five other genes identified in WGCNA and PPI networks were highly related to progression and poor prognosis of ccRCC [56]. However, they did not validate the genes in any experiment. Studies on BUB1B in ccRCC are rare. Although a study reported that along with 31 other genes, the four genes that we screened out were related to metastasis of ccRCC [57], it still had no validation. In our study, we screened UBE2C, BUB1B, RRM2, and TPX2 as key hub genes of ccRCC by integrated bioinformatics analysis. Experimental validation of RNA and protein levels showed that they were higher in cancerous than in paracancerous tissues. Overexpression of the four genes was also associated with an advanced clinical stage and poor overall survival rate. Thus, the neoteric key hub genes discovered in our study are likely to become a group of potential therapeutic targets of ccRCC.

Compared with other studies of ccRCC, our study is innovative in several aspects. Integrated bioinformatics analyses were applied to shed light on the relationship between DEGs and tumor clinical features in ccRCC. Moreover, we combined two gene expression microarray datasets from the GEO database and one RNA-seq dataset with corresponding clinical information from TCGA to screen out tumor stage-relevant DEGs of ccRCC. Moreover, the intersection of hub genes selected from key modules of WGCNA and DEGs selected from GEO datasets helped us identify 4 highly believable and accurate genes, which further validated the relevance between the genes and the progression and prognosis in ccRCC by multiple methods. However, a limitation of our approach is that further biological experiments on such aspects need to be carried out to verify the function of these potential biomarkers.

## 5. Conclusions

In conclusion, the present study identified key hub genes associated with the clinical stage and overall survival of ccRCC patients. Our study offers a deeper understanding of the molecular mechanisms associated with the development of ccRCC and provides potential biomarkers for early diagnosis and individualized treatment of patients at different stages of ccRCC.

## Figures and Tables

**Table 1:** Real-time PCR primers.

Gene	Real-time PCR primer sequences
UBE2C	F: 5′- AGTGGCTACCCTTACAATGCG -3′
	R: 5′- TTACCCTGGGTGTCCACGTT -3′
BUB1B	F: 5′- AAATGACCCTCTGGATGTTTGG -3′
	R: 5′- GCATAAACGCCCTAATTTAAGCC -3′
RRM2	F: 5′- GTGGAGCGATTTAGCCAAGAA -3′
	R: 5′- CACAAGGCATCGTTTCAATGG -3′
TPX2	F: 5′- ATGGAACTGGAGGGCTTTTTC -3′
	R: 5′- TGTTGTCAACTGGTTTCAAAGGT -3′
*β*-Actin	F: 5′- ACTCTTCCAGCCTTCCTTCC -3′
	R: 5′- CGTCATACTCCTGCTTGCTG -3′

**Table 2:** GSE datasets involved in our study.

Group	Quantity	GSE datasets
Normal tissues	72	GSE53757
ccRCC stage I	62	GSE53757+GSE73731
ccRCC stage II	29	GSE53757+GSE73731
ccRCC stage III	41	GSE53757+GSE73731
ccRCC stage IV	57	GSE53757+GSE73731
Total	261	

Note: ccRCC: clear cell renal cell carcinoma; GEO: Gene Expression Omnibus; GSE: GEO series.

**Table 3:** Four key hub genes selected from among 500 DEGs and 21 common hub genes from GSE73731 and TCGA-KIRC data.

Gene symbol	Description	Log FC in stage I	Log FC in stage II	Log FC in stage III	Log FC in stage IV
UBE2C	Ubiquitin-conjugating enzyme E2C	2.92	2.60	3.68	3.76
BUB1B	BUB1 mitotic checkpoint serine/threonine kinase B	3.07	2.24	3.26	3.19
RRM2	Ribonucleotide reductase regulatory subunit M2	3.08	2.9	3.74	4.10
TPX2	TPX2 microtubule nucleation factor	2.59	2.51	3.14	3.50

**Table 4:** Histopathological characteristics of the ccRCC patients.

Patient	Sex	Age (years)	Pathology	Location	Tumor size (cm)	Stage
1	Male	65	ccRCC, partially PRCC	Left	12∗10.5	III
2	Male	80	ccRCC	Left	14.8∗9.8	IV
3	Female	58	ccRCC, partially PRCC	Left	7∗4∗2.5	I
4	Male	63	ccRCC	Right	4.5∗4.5	IV
5	Female	81	ccRCC, partially PRCC	Left	3∗2.2	I
6	Female	56	ccRCC	Right	2.5∗3.1	IV
7	Male	57	ccRCC	Right	5∗4.5	II
8	Male	34	ccRCC	Right	3.5∗3.2	I

Note: ccRCC: clear cell renal cell carcinoma; PRCC: papillary renal cell carcinoma.

**Table 5:** IHC staining characteristics of hub genes from the Human Protein Atlas database.

Gene	Patient ID	Gender	Age (years)	Staining	Intensity	Quantity	Location
Normal tissues							
UBE2C	3229	Male	59	Medium	Moderate	>75%	Cytoplasmic Membranous Nuclear
RRM2	1859	Male	61	Not detected	Negative	None	None
TPX2	1933	Female	56	Low	Weak	25-75%	Cytoplasmic Membranous
Cancerous tissues							
UBE2C	2540	Male	61	High	Strong	>75%	Cytoplasmic Membranous Nuclear
RRM2	3616	Female	63	Not detected	Negative	None	None
TPX2	1831	Male	77	Medium	Strong	25%	Nuclear

Note: IHC: immunohistochemistry.

## Data Availability

All data included in this study are available upon request by contact with the corresponding author.
